# Epiglottal involvement of mycosis fungoides

**DOI:** 10.1016/j.jdcr.2025.01.025

**Published:** 2025-02-21

**Authors:** Margaret G. Mercante, Emily G. Tocco, Jennifer DeSimone

**Affiliations:** a University of Virginia School of Medicine, Charlottesville, Virginia; b Department of Dermatology, INOVA Medical Center, Fairfax, Virginia

**Keywords:** clinicopathological correlation, cutaneous oncology, cutaneous T cell lymphoma, dermatology, mycosis fungoides

## Introduction

Cutaneous T cell lymphomas (CTCLs) are a subtype of extranodal non-Hodgkin lymphomas that differ clinically from systemic lymphoma. The most common type of CTCL is mycosis fungoides (MF), a cutaneous lymphoma originating from epitheliotropic skin-resident effector memory T-cells.^1^ MF classically presents clinically with the appearance of patches, plaques, and tumors on nonsun-exposed skin.^2^ The pathologic features for diagnosis include mature T-helper memory phenotype, CD3+/CD4+/CD8−/CD45RO+, with monoclonal T-cell receptor gene rearrangements.^2^ While several clinical variants of MF have been described in the literature, they often present with consistent pathologic features.^3^

Here, we report an unusual presentation of MF with epiglottal involvement. We suggest that the challenge of diagnosing MF with clinical variants can be overcome with clinicopathological correlation. It is important for clinicians to understand the pathological features of MF to recognize unusual variants such as this case of epiglottal involvement, which can risk airway compromise with disease progression.

## Case report

**Fig 1:**
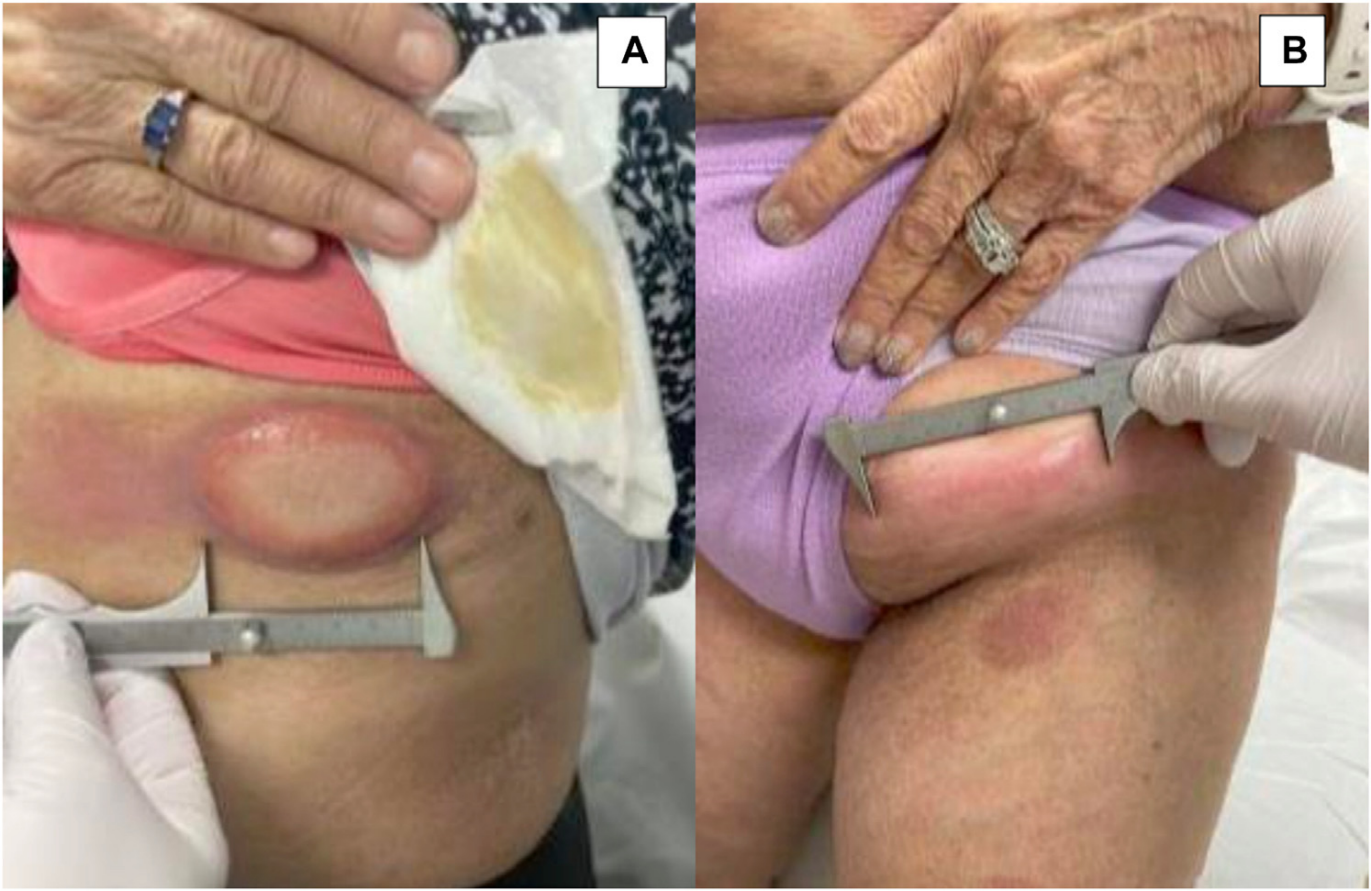
**A,** Clinical images show various mycosis fungoides lesions, including a prominent tumor on the flank and (**B**) an inguinal nodal tumor measured to be at least 9 centimeters wide.

A 66-year-old female presented to the multidisciplinary clinic diagnosed with stage 4 A2 (T3, *N*3, M0, B1) CTCL, MF type. She initially presented in 2022 with approximately 15% body surface area involved with patches and thin plaques. The patient was started on brentuximab in February of 2022, but she required several dose reductions and prolonged infusions over the course of 7 cycles. She progressed in disease in September 2022, experiencing a rapid progression to bulky nodal disease, including a 9-centimeter inguinal nodal tumor (Fig 1). Doxorubicin was started in October and quickly discontinued due to severe infusion reaction and intolerance. Three weeks later, gemcitabine was administered over the course of 7 cycles from October 2022 until January of 2023. Although she noted some improvement in her inguinal tumor, she experienced numerous drug side effects, including fever, dehydration, fatigue, and 30-pound weight loss. The patient completed local radiation to a large tumor on her left flank and back in February with good response, and she also initiated phototherapy at the same time, narrowband ultraviolet B therapy three times a week. She experienced rapid development of recurrent thick plaques in April 2023, and she was treated with fourth-line pralatrexate. Treatment was discontinued due to lack of response and severe diarrhea. She attempted to restart gemcitabine but was unable to tolerate it due to the development of severe noninfectious diarrhea and fever, leading to its discontinuation. Local radiation to large nodal masses in the left groin and axilla in September 2023 proved effective, and she was treated in October with pembrolizumab with excellent control of nodal disease and focal tumors as well as good drug tolerance. However, despite treatment, she continued to develop cutaneous tumors on the posterior leg in February 2024 and left flank in March 2024.

**Fig 2:**
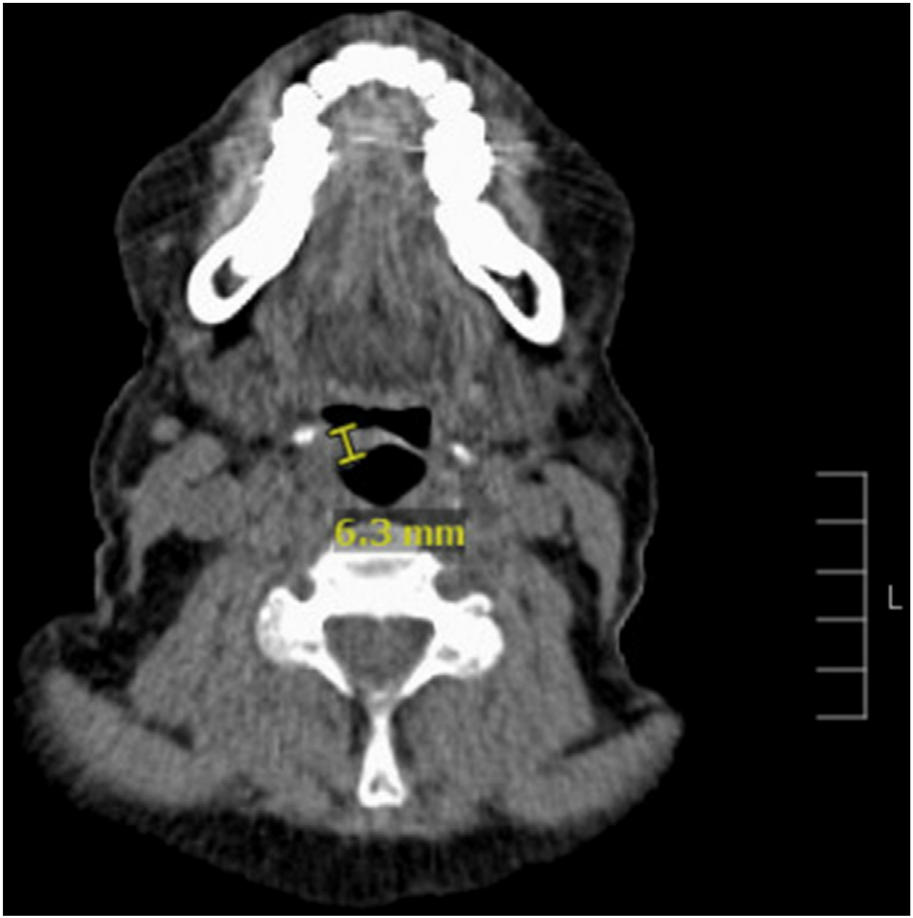
Axial computed tomography scan of oropharynx demonstrating a well-defined supraglottic mass (*yellow marker*) 6.3 millimeters in width, revealed to be epiglottic involvement of mycosis fungoides.

The patient experienced sudden-onset dysphagia in late March of 2024, 5 months into her course of pembrolizumab treatment. She reported throat tightness and soreness, initially thought to be related to infection or allergies. After her eighth infusion of pembrolizumab, her dysphagia significantly worsened, and she could not swallow solids. She presented to urgent care twice and received 2 courses of antibiotics with no symptomatic improvement. The patient was referred to the emergency department, where a computed tomography scan was obtained to assess airway blockage. Imaging revealed a supraglottic mass with complete effacement of the airway (Fig 2). Otolaryngology initiated a scope, which confirmed a supraglottic mass with ulceration. She was started on 60 milligrams of oral prednisone for airway protection and control of epiglottic edema with significant improvement in symptoms.

Initial biopsy of the epiglottic mass revealed nondiagnostic inflamed squamous atypia. The possibility of an undiagnosed early-stage mucosal squamous cell carcinoma with tumor-infiltrating lymphocyte response on programmed cell death protein-1 inhibitor therapy was considered. A repeat biopsy of the mass revealed a dense lymphocytic infiltrate composed of atypical CD4+ lymphocytes with loss of CD7 and T-cell gene rearrangement with clonal sequence identical to nodal and skin clones, consistent with epiglottic involvement of MF. Notably, large cell transformation was present on biopsy.

She was continued on prednisone and simultaneously treated with 30 Gy of targeted radiation therapy over 15 fractions, with rapid improvement and resolution of dysphagia.

## Discussion

Although patients with MF typically present with cutaneous involvement, clinicians should be aware of the potential to spread to viscera, particularly in more advanced disease. Epiglottal involvement in MF has rarely been documented in literature, including MF manifestation as a tumor of the arytenoid cartilage and epiglottis.^4^ Other rare oral and upper respiratory manifestations of CTCL, including laryngeal involvement, have also been infrequently reported.^5^^,^^6^ These patients with visceral involvement typically have advanced stage MF, such as in this case, and present with a primary complaint of painless dysphagia. This case contributes to current literature on visceral involvement of MF by noting the possible risk of airway compromise in patients, along with describing successful treatment of epiglottal involvement for a patient with large cell transformation and aggressive disease using targeted radiation therapy. Successful treatment of the patient’s visceral MF involvement with radiation highlights an effective therapeutic approach and offers clinicians valuable insights for managing unusual presentations of MF. Clinicians should be aware of this rare manifestation of CTCL and the important implications of the disease spreading to the epiglottis.

## Conflicts of interest

None disclosed.

## References

[1] Vaidya T., Badri T. (2024). StatPearls [Internet].

[2] Willemze R., Jaffe E.S., Burg G. (2005). WHO-EORTC classification for cutaneous lymphomas. Blood.

[3] Virmani P., Myskowski P.L., Pulitzer M. (2016). Unusual variants of mycosis fungoides. Diagn Histopathol.

[4] Hood A.F., Mark G.J., Hunt J.V. (1979). Laryngeal mycosis fungoides. Cancer.

[5] Goggins C.A., Gocke M.T., Jang S., DeSimone J.A. (2019). Oral mycosis fungoides with CD30+ large cell transformation successfully treated with brentuximab vedotin. JAAD case reports.

[6] Bauman T.M., Wichterman C.M., Musiek A.C., Nemer K.M. (2017). Hoarseness as a presentation of mycosis fungoides infiltrating the larynx. BMJ Case Rep.

